# Role of Meconium and Hypoxia in Meconium Aspiration-Induced Lung Injury in Neonatal Rabbits

**DOI:** 10.1155/2010/204831

**Published:** 2010-12-30

**Authors:** Alex Zagariya, Monica Sierzputovska, Shan Navale, Dharmapuri Vidyasagar

**Affiliations:** ^1^Neonatology Research Laboratories, Department of Pediatrics, Michael Reese Hospital, Chicago, IL 60616, USA; ^2^Division of Neonatology, Department of Pediatrics, The University of Illinois, Chicago, IL 60612-7324, USA; ^3^Poznan Childrens Hospital, Poznan, Poland

## Abstract

*Background*. We previously showed that meconium cuases lung cell death by apoptosis and inflammatory cytokine expression. Whether this is due to meconium exposure itself, or meconium related hypoxia remains unclear. *Objectives*. To elucidate the effects of meconium, saline, milk, hypoxia and hyperoxia induced lung injury. *Design/Methods*. We studied 5 groups of rabbit pups: (I) normal saline; (II) Milk; (III) 10% solution of meconium; (IV) only to 15 minutes of hypoxia (10% O_2_), and (V) 5 minutes of hypoxia (95% O_2_). After exposure lung lavage cells were used for apoptotic cell count and cytokine expression. *In vitro* response of human A 549 epithelial cells to meconium-and milk exposure was also studied. *Results*. There was no difference in cell death between saline and milk groups. However, meconium caused a significant cell loss compared to saline and milk—Inflammatory cytokines increased significantly in meconium group compared to saline or milk group. Although hypoxic and hyperoxic lungs showed increased inflammatory reaction compared to saline-treated lungs, this injury was not significant compared to meconium group. Studies with A549 cells also showed similar results. *Conclusions*. We conclude that lung cell injury in meconium aspiration is maily from meconium itself.

## 1. Introduction

In previous studies, we demonstrated that meconium exposure in neonatal rabbit lungs leads to significant cell death and expression of inflammatory cytokines [1]. It can induce compliment activation, apoptosis, and phospholipase A_2_ activation [2]. In clinical situation, the most deleterious effect of MAS is the development of both increased pulmonary vascular resistance (PVR) and associated pulmonary hypertension [3]. The cause of increased PVR is believed to be related primarily to hypoxia and by the meconium-induced lung injury; however, the relative contribution of other mechanisms and mediators are unknown [4]. Newborn infants with meconium aspiration frequently require a high concentration of oxygen. High oxygen exposure is also associated with adverse pulmonary effects [5, 6].

In clinical meconium aspiration syndrome (MAS), there are three major mechanisms of lung cell injury. (1) Meconium and its components may by itself cause cell injury. (2) Associated hypoxia can also cause cell injury. (3) During treatment, lungs are exposed to high oxygen concentration, which may also cause cellular injury. Thus, all these mechanisms may play a major role in meconium-induced lung cell apoptosis and increased cytokine expression. However, it is not known whether hypoxia and hyperoxia will independently induce lung injury similar to meconium.

We hypothesized that lung injury associated with meconium may be due to cellular responses to hypoxia and hyperoxia seen in MAS. In order to test our hypothesis, we designed a study to determine the independent effects of meconium, hypoxia, and hyperoxia and inert substances such as saline and milk in inducing lung cell apoptosis and inflammatory cytokine response. In addition to the above studies, we conducted in vitro experiments using A549 cells to study the independent effect of meconium and saline in the presence of hypoxia and hyperoxia in inducing apoptotic cell death. In this paper, we present our findings of these studies.

## 2. Material and Methods

### 2.1. Meconium Preparation

Fifteen first-pass human meconium samples were obtained from full-term, healthy neonates. One g of fresh newborn infant meconium was homogenized on ice in a blender with 9 ml of 0.9% NaCl to a 10% (weight/volume) final concentration followed by spin down at 5.000 RPM for 20 min at 4°C to separate supernatant and pellet (debris). Then supernatant filtered via a glass filter followed by sterilization via 0.2 m filter (both filters were from Millipore Co., Bedford, MA) and used for instillation in the lungs. Meconium of higher then 10% concentration in our experiments caused death of many rabbit pups. Their breathing was slower, followed by complete apnea. Because of this reason, we decided to use a 10% meconium solution for our studies.

### 2.2. Animal Model

We used two-week-old New Zealand white rabbit pups (LSP Industries, Union Grove, WI) as animal models for our study [1]. The animals were cared for and handled according to National Institute of Health guidelines. The Animal Care and Use Committee at Michael Reese Hospital, Chicago, Illinois, approved this study. For a few days preceding the experiment, animals were housed with their mother in stainless steel rabbit cages. The mothers were fed with regular Purina rabbit chow (Scientific Animal Feed Co., Arlington Heights, IL).

We used six rabbits in each group and for each time period (the minimum allowing for obtaining statistically significant data). Five groups of animals were studied: Group I: saline-instilled (subgroup a: only saline; subgroup b: saline in hypoxia; subgroup c: saline in hyperoxia); Group II: exposed to hypoxia (FiO_2_: 10%) for a period of 15 minutes; Group III: exposed to hyperoxia (FiO_2_: 95%) for a period of 15 minutes; Group IV: instilled with 10% meconium solution (subgroup a: only meconium; subgroup b: meconium in hypoxia; subgroup c: meconium in hyperoxia); Group V: instilled with milk “Similac” with iron, infant formula, Ross Pediatrics. Saline, milk, or meconium was instilled in the volume of 300 g per 200 g of body weight. Animals did not tolerate hypoxia of FiO_2_ less than 10% for longer than 15 minutes. The groups subjected to saline, meconium, and milk instillation (Groups I, IV, and V) were studied at 0-, 4-, 8-, and 24-hour time periods from insult and then they were sacrificed. Before instillation, we anesthetized rabbits with 10 mg/kg Ketamine and 1 mg/kg Xylazine, given intraperitoneally [1]. A small midline incision was made on the ventral aspect of the neck to expose the trachea; saline, meconium, or milk was then instilled into lungs followed by 5 ml of bolus of air to spread meconium into the lungs. The skin incision was closed with 4–0 nylon suture; the rabbits were allowed to breathe room air spontaneously for 0, 4, 8, and 24 hours and then were euthanized with Nembutal (100 mg/kg, i.p.). Immediately after sacrifice, the chest was opened by a midline incision, lungs were isolated and lung lavage was obtained as described below. We evaluated a timeline for the rabbits to develop signs of lung injury rather than test for their survival rate at the end of experiment.

After the sacrifice of each animal and isolation of the lungs, the tracheas were cannulated and lavage was performed through the trachea and main bronchi to recover lung cells as described earlier [1]. We collected lung lavage by 10 ml aliquot three times to collect 30 ml of lavage fluid per rabbit studies. We obtained a total protein after several times cell sonication using an ultra sound sonicator (and then spinned down at 10,000 RPM for 10 minutes to isolate cell debris. Then we precipitated supernatant-contained protein by acetone and then pellet dissolved in 50 *μ*L of ddH_2_O to measure protein concentration by Bradford Method. Finally, we added 50 *μ*L of SDS sample buffer to the sample and performed 10% SDS gel electrophoresis.

### 2.3. Cell Death Determination

Lung lavage fluid was spun down at 5,000 RPM for 10 min at 4°C. Then supernatant was discarded and cell pellet then was stained with 0.1 g/ml Ethidium Bromide and Acridine Orange (Sigma Chemical Co., St. Louis, MO). Fluorescent microscopy of the lavage cells (mostly macrophages, leukocytes and neutrophils) was performed as described by us earlier [1]. Fluorescent analysis clearly differentiates live cells from dead. Live cells (stained with Acridine Orange) appeared green, while dead cells (stained with Ethidium Bromide) appeared red. The number of dead cells was counted three times, in different areas of the slide, and the results were calculated as a percentage from each 100 cells counted.

### 2.4. Studies of Cytokines Proteins Using ELISA

The supernatants obtained after centrifugation of the samples were used for ELISA assays as described earlier [1]. We measured the IL-1*β*, IL-6, IL-10, and TNF*α* cytokine expressions in pictograms in milliliter (pg/ml) using commercially available ELISA kits as described by their manufacturer (R&D Systems Co., Minneapolis, MI).

In a separate experiment, independent effects of hypoxia or hyperoxia on meconium-induced lung injury were studied using human lung epithelial A549 cells in culture. Cells were treated in hypoxic or hyperoxic conditions and then 10% meconium solution was added. Detailed description of cell culture work is described below.

### 2.5. Cell Culture

We are conducting in vitro studies to demonstrate meconium-induced inflammation using cultured A549 cells. The human lung adenocarcinoma cell line A549 was obtained from American Type Culture Collection and cultured in Ham's F12 medium supplemented with 10% fetal bovine serum (FBS). Cells collected were over 90% pure as assessed by acridine orange staining. All cells were seeded in 24-well or 6-well chambers and all experiments were conducted at densities of 80%–90% in a serum-free Ham's F12 medium. Cells were treated first in hypoxia (or hyperoxia) for 15 minutes, and then 10% meconium was added to the plates and incubated for eight hrs in hypoxic or hyperoxic conditions at 37°C before harvesting. For quantitation of cell death, we used Acrydine Orange (live cell staining, green color) and Ethidium Bromide (dead cell staining, orange color) stained cells.

### 2.6. Data Analysis

All measurements were compared using the repeated measure analysis of variance. Results are mean ± standard deviations of at least four experiments. In morphological experiments, 300 cells were counted per each slide and numbers were compared between the groups. Paired evaluations were made for experimental and control conditions within each experiment, and significance was determined by student's *t*-test. Statistical significance was determined using ANOVA with *P* < .05 regarded as significantly important. All statistics was calculated by InStat Biostatistics software.

## 3. Results

Saline-instilled animals (control group) revealed approximately same amount of lavage cells as milk-instilled lungs.  Table 1(a) shows cell count in lung lavage at different times.

Meconium-instilled animals demonstrated significantly lower lavage cell number at a period of 8 hrs after meconium instillation versus milk- or saline-instilled (10.7 ± 5.2 × 10^3^ versus 15.2 ± 3.7 × 10^3^ versus 14.2 ± 2.1 × 10^3^) and 24 hrs (8.8 ± 11.1 × 10^3^ versus 13.8 ± 2.6 × 10^3^ versus 12.9 ± 10^3^). Hypoxia was associated with low cell count at 15 minutes treatment time similar to meconium. Hyperoxia showed no differences (Table 1(b)).

**Figure 1:**
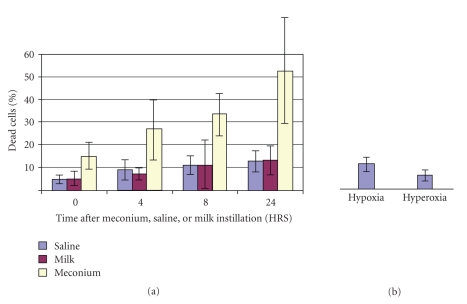
Cell death in groups of animals instilled with saline, milk and 10% meconium. (a) shows percent of dead cells at different time periods after instillation. The number of dead cells after meconium instillation was significantly higher than after saline and milk instillation at all time periods studied (*P* < .05). (b) shows lavage cell death in animals treated with hypoxia or hyperoxia. There was no statistical difference in the percent of dead cells between hypoxic or hyperoxic conditions. These numbers were negligibly small and comparable to saline-instilled animals.

Observed cell death in lung lavage can be very significant and can reach up to 52% within 24 hrs after meconium instillation [1].  Figure 1(a) demonstrated presence of lavage cell death in meconium, saline, and milk. There were no significant differences between these groups. Cell death in hyperoxic and hypoxic conditions are shown in Figure 1(b). The number of dead cells after meconium instillation was significantly higher than after saline and milk instillation and the differences were statistically significant (*P* < .05). Cell death significantly increased with time in the meconium group, but not in the saline or milk groups (Figure 1(a)). Hypoxia resulted in 7.1% ± 1.3% and hyperoxia in 4.4% ± 0.5% of dead cells in Figure 1(b). 

On microscopic examinations we observed many inflammatory infiltrated cells and many opened alveoli. Arterial walls were thick compared to saline-instilled animals. We also observed fragmented basement membranes, which also were infiltrated with inflammatory cells. Hypoxia also increases the size of alveolar septae, which shows broken alveoli (not shown).

The effect of hyperoxia was not significant in saline as well as meconium-instilled lungs. Hyperoxic animals demonstrated sleepy behavior and its effect was seen already after 15 seconds after treatment. Both hyperoxia and hypoxia animals demonstrated signs of respiratory distress (retraction, nasal flaring). Regardless of the noted effect of hypoxia or hyperoxia-induced damage, meconium-induced damage was significantly more destructive and meconium-induced lung injury was significantly higher than in other groups studied.

Meconium-instilled animals demonstrated increase of total protein content in the lung lavage (see Table 2(a)). Meconium-induced protein levels were significantly higher compared to levels observed in lung lavage from animals instilled by saline, milk as well as animals exposed to hypoxic or hyperoxic conditions (Table 2(b)). In meconium-treated lungs, 6.2 ± 1.7 mg/ml of total protein was detected 24 hrs after meconium instillation; 0.24 ± 0.2 mg/ml in saline-instilled lungs; 2.0 ± 0.4 mg/ml in milk-treated lungs; 2.3 ± 0.9 mg/ml in hypoxic lungs; 3.1 ± 1.1 mg/ml in hyperoxic lungs. Data for meconium-instilled lungs was statistically significant versus all other groups. Data for hyperoxic lungs also was statistically significant versus the saline group (*P* < .05) and not significant versus the hypoxic group (*P* > .05).

Intratracheal instillation of meconium causes the dramatic expression of inflammatory cytokines in lung lavage but not in saline- or milk-instilled lungs. Saline-exposed newborn rabbit lung lavage cytokine levels were similar at 4, 8, and 24 hrs. In this study, we demonstrated expression of TNF*α*, IL-1*β*, IL6, and IL10 cytokines in different study groups of rabbits. The TNF*α* and IL-6 inflammatory cytokines were expressed significantly in meconium-instilled lungs (Table 3(a)).

Both of these cytokines were expressed about four times higher compared to saline-treated than in lavage-treated lungs. Milk-instilled animals did not show any significant expression of discussed cytokines versus saline-instilled, and milk-instilled and saline-instilled animals showed approximately similar, not significant levels of TNF*α* and IL-6 cytokine expression. Surprisingly, IL-1*β* was expressed about twofold higher 4 hrs after meconium-instillation (maximal observed expression in IL-1*β*) than saline-instilled lungs (see Table 3(a) for details).

In hypoxic or hyperoxic conditions, the levels of inflammatory cytokines TNF*α* and IL6 were increased significantly (Table 3(b)). Anti-inflammatory cytokine IL-10 levels were not significant in any of the groups studied.

### 3.1. Results of A549 Cell Studies

**Figure 2:**
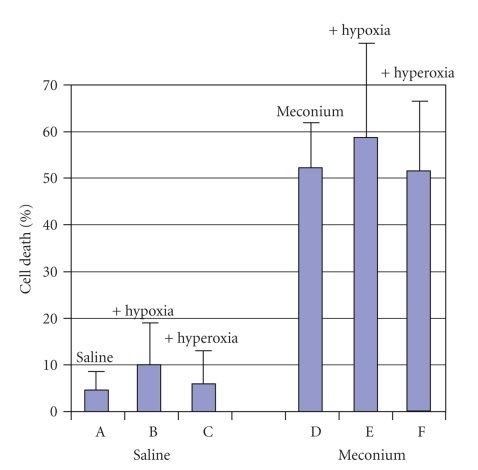
Cell death in A549 cell line studies. Percent of cell in saline- (A, B, C) or meconium- (D, E, F) treated A549 cells in presence of hypoxia (B, E) or hyperoxia (C, F). Meconium + hypoxia (E) or hyperoxia (F) demonstrated some increase in cell death compared to saline + hypoxia (B) or hyperoxia (C). *P* < .05 meconium (D, E, F) versus saline (A, B, C).

We found that meconium-treated cells similar to lung lavage cells demonstrated dramatically higher percent of cell death (Figure 2, D, E, and F) compared to saline-treated cells (Figure 2, A, B, and C). This difference was statistically significant (*P* < .05). We also found that hypoxia + meconium group showed some increase in number of cell death and inflammatory cytokines expression compared to hyperoxia + meconium group; however, this increase was not significant (*P* > .05).

## 4. Discussion

Meconium aspiration syndrome is frequently associated with hypoxia. Hypoxia is accompanied with mechanisms responsible for clinical manifestations of MAS [7]. Levels of hypoxia contribute to the inflammation induced by meconium as noted by increased cytokine production in hypoxic conditions. Leeper-Woodford and Detmer showed that acute hypoxia in lungs may induce enhanced NF-kB activation in alveolar macrophages, resulting in the increased production and release of inflammatory cytokines such as TNF*α* [4].

Hyperoxia has also been reported to cause pulmonary pathology and dysfunction, leading to death in several species [8, 9]. In survival studies, Frank et al. [8] showed that newborn lungs are particularly susceptible to hyperoxic exposure because of their inability to increase protective lung antioxidant enzymes, resulting in 50% mortality rate by three days. Kelly et al. [10] reported a mortality rate of 21% in newborn exposed to 95% hyperoxia for 96 hrs. Hyperoxia is associated with low inflammatory cell levels; protease activity and levels of total protein were slightly increased compared to that of saline-instilled animals, suggesting a similar degree of pulmonary dysfunction. This dysfunction may be due to lung inflammation and capillary leaks. Cell death in lungs may be induced by numerous factors, the most critical of them is meconium and hyperoxia [5, 7, 11–16]. Previous studies have described in detail the effects of hyperoxia on the lungs in different animal models. Buckley et al. examined cell death by apoptosis in *ex vivo *cell culture from hyperoxia-exposed rat lungs [11]. Hagimoto et al. showed Fas/Fas L pathway in apoptosis mediation in mice [17]. Holopainen et al. used piglet model to show early necrotic changes in lung epithelium [12].

Newborn rabbits cannot tolerate hypoxic or hyperoxic conditions more than 15 minutes. Many of them are dying. However, we realize that hypoxia presents in lungs during all time in MAS. Interestingly, those human lung epithelial cells in culture were able to survive in hypoxic and hyperoxic conditions for much longer time. That is why we used a cell culture for studies in addition to a newborn rabbit model. 

Hyperoxia is a well-known inflammatory factor, causing severe response in the lungs. It is of specific interest because of the wide use of mechanical ventilation and oxygen treatment in the management of MAS. Allen et al. [18] used cultured respiratory epithelial-like cells treated with hyperoxia alone, TNF*α* alone, or both factors to show their effects on IL-8 gene expression. He demonstrated that hyperoxia alone has a minimal effect on IL-8 expression, but synergistically increases it in the presence of TNF*α*. Ben-Ari et al. base their studies on neonatal rats, showing that the exposure of lungs to 100% oxygen causes the production of pulmonary TNF*α* on the third day and IL-6 on days 6–9 of hyperoxia [19, 20].

There is a similar degree of pulmonary dysfunction in hypoxic and hyperoxic animals. In our studies, meconium-instilled animals demonstrated significantly higher levels of cell death and higher cytokine expression, compared to other treatments. Hypoxic and hyperoxic animals produce initial lung inflammation (polymorphonuclear cells and inflammatory cytokines increase).

We compared the effects of milk instillation to meconium instillation studies as reported before [15]. To the best of our knowledge, there is no data about the effects of milk instillation on lung cell apoptosis or cytokine expression in the literature available, making our study unique in this area. The effects of milk alone on lung were comparable to the effects of saline.

Finally, another manifestation of lung injury is capillary leak resulting in an increase of total protein concentration in lavage fluid, which was high in meconium group (Table 3). In contrast, the total amount of protein in BAL in milk-instilled animals was comparable to saline-instilled animals.

In the present study, we compared the inflammatory effect of meconium with effects of milk instillation. We also presented a data about the effect of hypoxia and hyperoxia on lung cell death and expression of inflammatory cytokines.

Our data seems to prove that none of the irritants causing acute lung injury contributed to lung cell death or cytokine expression to the extent seen by the aspiration of meconium. Hypoxic and hyperoxic insults, even if treated for only 15 minutes, resulted in minimal changes, whereas milk and saline caused even less injurious effect. Hypoxia, always a factor in meconium-induced lung injury, does not seem to be responsible for its injury and apoptotic effect. More studies are needed to find the role of mechanical stress of ventilation or the characteristics of meconium such as particle size, viscosity, and so forth on lung cell injury, including a longer time of treatment, as 2, 4, 8, and 24 hrs of hypoxia or hyperoxia treatment.

In summary, we demonstrated that hypoxia and hyperoxia resulted in no significant increase in cell death and inflammation in newborn rabbit pups. An effect of milk exposure was comparable to saline effects. The effects described above were much smaller and more delayed compared to meconium effect. Our present studies demonstrate that aspiration of milk and saline into the lungs will not cause a significant lung injury.

Overall, our data demonstrate that meconium-induced lung injury may not be explained by the concomitant presence of milk, hypoxia, or hyperoxia in MAS. Meconium with its multiple compounds is likely the major agent, which induces lung inflammation and lung cell death.

## Figures and Tables

**Table 1a:** (a) Total lung lavage cell count after exposure to saline, meconium, and milk.

Time	Saline (total lavage cells)	Meconium (total lavage cells)	Milk (total lavage cells)
0 hours	15.1 ± 2.1 × 10^3^	14.8 ± 5.1 × 10^3^	16.2 ± 3.2 × 10^3^
4 hours	16.0 ± 2.7 × 10^3^	12.1 ± 8.8 × 10^3^	14.7 ± 2.9 × 10^3^
8 hours	14.2 ± 2.1 × 10^3^	10.7 ± 5.2 × 10^3^*	15.2 ± 3.7 × 10^3^
24 hours	12.9 ± 2.4 × 10^3^	8.8 ± 11.1 × 10^3^*	13.8 ± 2.6 × 10^3^

*P < .05 versus 0 hrs.

Meconium group demonstrated a lower total lavage cell number compared to saline and milk groups. At 8 and 24 hrs after meconium instillation, the decrease of cells is significantly lower (*P* < .05) than baseline.

**Table 1b:** (b) Total lung lavage cell count after exposure to hypoxia and hyperoxia.

Time	Hypoxia group (total lavage cells)	Hyperoxia group (total lavage cells)
15 min	9.12.3 × 10^3^	12.62.5 × 10^3^

Lavage from hypoxic animals contained lower number of total cells, compared to hyperoxia group.

**Table 2a:** (a) Total protein content in lung lavage fluid after exposure to saline, meconium, and milk (mg/ml).

Time	Saline group (total protein, mg/ml)	Meconium group (total protein, mg/ml)	Milk group (total protein, mg/ml)
0 hours	0.09 ± 0.1	0.9 ± 0.2	1.2 ± 0.2
4 hours	0.11 ± 0.1	4.1 ± 1.0*	1.1 ± 0.7
8 hours	0.20 ± 0.2	5.7 ± 2.1*	1.7 ± 0.2
24 hours	0.24 ± 0.2	6.2% ± 1.7*	2.0 ± 0.4

Total protein in lung lavage increased significantly in meconium group compared to both saline and milk groups (*P < .05). There was a 10–20-fold increase in protein content meconium group compared to saline group and about 4-folds compared to milk group. Instillation of milk resulted in about 8–10-fold increase of total protein content compared to saline (*P < .05). Please note that values at 0 hr indicate preinstillation baseline values in each group.

**Table 2b:** (b) Total protein levels after in hypoxic or hyperoxic conditions in lung lavage (mg/ml).

Time	Hypoxia group (total protein, mg/ml)	Hyperoxia group (total protein, mg/ml)
15 min	2.3 ± 0.9*	3.1 ± 1.1*

There was no difference in protein content between hypoxia and hyperoxia group. However, these levels were significantly lower than in the meconium group (*P* < .05) (see above in Table 2(a)) and higher than the saline group.

**Table 3a:** (a) Expression of TNF*α*, IL-1*β*, IL-6, and IL-10 cytokines after saline, meconium, or milk instillation (in pg/ml).

	Sal 8 hrs	Mec 4 hrs	Mec 8 hrs	Mec 24 hrs	Milk 4 hrs	Milk 8 hrs	Milk 24 hrs
TNF*α* (*P* < .05)	0.39 ± 0.11	0.80 ± 0.32	1.45 ± 0.84	1.24 ± 0.42	0.40 ± 0.11	0.43 ± 0.21	0.64 ± 0.18
IL-1*β*	0.29 ± 0.19	0.44 ± 0.22	0.33 ± 0.15	0.25 ± 0.12	0.12 ± 0.05	0.17 ± 0.05	0.65 ± 0.12
IL-6 (*P* < .05)	0.21 ± 0.14	0.44 ± 0.25	0.92 ± 0.34	0.81 ± 0.26	0.24 ± 0.05	0.19 ± 0.10	0.68 ± 0.12
IL-10	0.25 ± 0.09	0.23 ± 0.12	0.21 ± 0.17	0.27 ± 0.19	0.31 ± 0.27	0.16 ± 0.17	0.28 ± 0.14

Changes in cytokine levels within the saline-treated or milk-treated groups were not significant. In meconium group, cytokine levels increased significantly at 8 and 24 hrs.

**Table 3b:** (b) Expression of TNF*α*, IL-1*β*, IL-6, and IL-10 cytokines after 15-minute treatment in hypoxic or hyperoxic conditions.

Cytokines (pg/ml)	Saline 8 hrs	Hypoxia 15 min	Hyperoxia 15 min
TNF*α* (*P* < .05)	0.39 ± 0.11	0.54 ± 0.29	0.71 ± 0.21
IL-1*β*	0.29 ± 0.19	0.44 ± 0.15	0.40 ± 0.17
IL-6 (*P* < .05)	0.21 ± 0.14	0.69 ± 0.20	0.67 ± 0.36
IL-10	0.35 ± 0.19	0.43 ± 0.39	0.39 ± 0.20

Cytokine levels in hypoxic or hyperoxic conditions were elevated. However, a significant increase was observed only for TNF*α* and IL6 expressions for both hypoxic and hyperoxic groups compared to saline-instilled group.
